# New Insight into the SAR of Pyrimido [4,5-b][1,4] Benzothiazines as 15-lipoxygenase Inhibitors

**Published:** 2013-06

**Authors:** Nona Pooryaghoobi, Mehdi Bakavoli, Maliheh Alimardani, Tahmineh Bazzazan, Hamid Sadeghian

**Affiliations:** 1 Department of Chemistry, Mashhad Branch, Islamic Azad University, Mashhad, IR Iran; 2 Department of Chemistry, School of Sciences, Ferdowsi University of Mashhad, Mashhad, 917751436, IR Iran; 3 Student Research Committee, Department of Laboratory Sciences, School of Paramedical Sciences, Mashhad University of Medical Sciences, Mashhad, IR Iran; 4 Antimicrobial Resistance Research Center, Mashhad University of Medical Sciences, Mashhad 91967-73117, IR Iran; 5 Department of Laboratory Sciences, School of Paramedical Sciences, Mashhad University of Medical Sciences, Mashhad, IR Iran

**Keywords:** DMAB, Docking, MBTH, Peroxide formation, SLO

## Abstract

***Objective(s):*** Recently we reported that the soybean 15-lipoxygenase (SLO) inhibitory activity of pyrimido[4,5-b][l,4]benzothiazines largely depends on the orientation of sulfur atom of thiazine core towards Fe^III^-OH in the active site pocket of the enzyme with subsequent oxidation of sulfur to sulfoxide. In this paper the results of a comparative study on the SLO inhibitory activities of the mentioned compounds using *ab initio* calculations and docking analyses has been reported.

***Materials and Methods:*** Structure optimization and docking analyses were performed using HyperChem 7.5 and AutoDock Tools 4.0 respectively. Enzyme assessment was reduced using spectrophotometric MBTH-DMAB method.

***Results*** *:* The inhibitory activity of synthetic 2-substituted pyrimido[4,5-b][l,4]benzothiazines against soybean 15-lipoxygenase (SLO) was evaluated and structure activity relationships and binding modes of their 4-H and 4-methyl analogs were studied using docking analysis and *ab initio* calculations.

***Discussion:*** The results of these studies showed that the lack of 4-methyl substituent in the pyrimido[4,5-b][1,4]benzothiazine molecules greatly reduces their lipoxygenase inhibitory activities and it was also found that the HOMO energy difference between the 4-H and 4-Methyl analogs can be responsible for the observed inhibitory activity reduction.

***Conclusion:*** Our molecular modeling studies shows that by using more flexible amino acids during the docking process, more rational results can be obtained. The method of measuring the lipoxygenase activity is also of prime importance for the study of structure activity relationship.

## Introduction

It is well documented that mammalian lipoxygenases (LO’s) are non-heme iron-containing enzymes responsible for the oxidation of polyunsaturated fatty acids and esters to hydroperoxy derivatives (1). These are heterogeneous families of enzymes distributed widely throughout the plant and animal kingdoms (2), and named according to the position at which a key substrate, arachidonic acid (AA), is oxidized. Among the mammalian lipoxygenases involved in the etiology of human disease, 5-lipoxygenase (5-LO) is now well established as a target for reducing the production of leukotrienes (important in particular asthma) (3, 4). More recently, 15-lipoxygenase (I5-LO) has emerged as an attractive target for therapeutic intervention (5). 15-LO has been implicated in the progression of certain cancers (6, 7) and chronic obstructive pulmonary disease (COPD) (6).

Evidence for the inhibition of 15-LO in the treatment of vascular disease is, however, most compelling (8). Both transgenic and knockout studies implicate a role for 15-LO in atherogenesis (9,10). The enzyme is abundantly expressed in macrophages residing within the atherosclerotic lesion (10). In addition, the immediate products of 15-LO oxidation of AA and linoleic acid (LA) have been shown to be pro-inflammatory (11) and pro-thrombotic (12).

It is also found that 15-LO is linked to cardiovascular complications since it is known to participate in oxidative modification of low-density lipoproteins (LDL) leading to the development of atherosclerosis (13).

Three different strategies have been developed to inhibit the LO’s pathway (12). They involve (i) redox inhibitors or antioxidants, which interfere with the redox cycle of 15-LO, (ii) iron-chelator agents, and (iii) non-redox competitive inhibitors, which compete with AA to bind the enzyme active site.

**Scheme 1:**
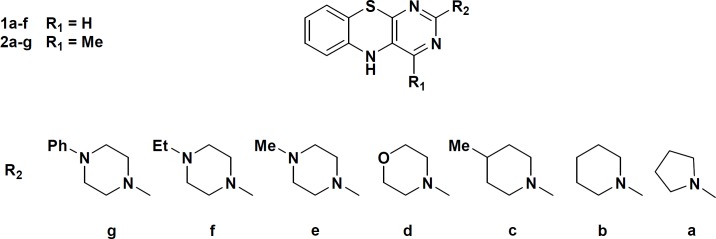
chemical structure of compounds 1a-f and 2a-f

Recently we reported the results of our studies on the soybean 15-lipoxygenase (SLO) inhibitory activities of some pyrimido[4,5-b][l,4]benzothiazines derivatives 1a-f and 2a-f and on the basis of the structure activity relationship (SAR) studies we suggested that the inhibitory activity of these molecules largely depends on the orientation of sulfur atom of thiazine core towards chelated Fe^III^-OH in the active site pocket of the enzyme with subsequent oxidation of sulfur to sulfoxide (14, 15). In this paper we wish to report the results of a comparative study on the SLO inhibitory activities of a group of pyrimido[4,5-b][l,4]benzothiazines and their 4-H and 4-methyl analogs (Scheme 1) using *ab initio* calculations and docking analysis. In the research other lipoxygenase inhibitory assessment in which the enzyme activity measurement was made is also used according to the reported peroxide formation protocols (16). In the other work the three dimensional structural requirements of some natural organosulfur compounds for SLO inhibitory activity using comparative molecular field analysis (CoMFA) and comparative molecular similarity indices analysis (CoMSIA) was studied (17).

## Materials and Methods

***Chemistry***

Compounds **1a-f** and **2a-f** were synthesized according to the procedure reported in the previous literatures by starting from uracil and 4-methyluracil as primary reagents (14, 15).

***Structure optimization***

Structures **1a-f** and **2a-f** were simulated in chem3D professional; Cambridge software (18); using MM2 method (RMS gradient = 0.1 kcal mol^-1^) (19, 20). In the second optimization, output files were minimized under Semiempirical AM1 methods (Convergence limit = 0.01; Iteration limit=50; RMS gradient = 0.1 kcal mol^-1^; Polak-Ribiere optimizer algorithm) then the output files were minimized under *ab initio* methods with 6-311G^*^ basis set (Convergence limit=le-5; Iteration limit=50; RMS gradient=0.1 kcal mol^-1^; Polak-Ribiere optimizer algorithm Hyper Chem7.5 (21). After geometry optimization and docking, single point properties of docked molecules such as energy of HOMO and LUMO were calculated using *ab initio* method with 6-311G^*^ basis set (convergence limit= le-5; iteration limit= 50). The initial guess of the MO coefficients is from eigenvectors of the core Hamiltonian in HyperChem 7.5 (21).

Crystal structure of soybean lipoxygenase-3 (arachidonic acid 15-lipoxygenase) complex with 13(S)-hydroproxy-9 (Z)-2,ll(E)-octadecadienoic acid was retrieved from RCSB Protein Data Bank (PDB entry: 1IK3). 

***Molecular docking***

Automated docking simulation was implemented to dock **1a-f** and **2a-f** into the active site of SLO with Auto Dock Tools version 4.2 (revision 30) (22) using Lamarckian genetic algorithm (23). This method has been previously shown to produce bonding models similar to the experimentally observed models (16, 24, 25). The torsion angles of the ligands were identified, hydrogens were added to the macromolecule, bond distances were edited and solvent parameters were added to the enzyme 3D structure. Partial atomic charges were then assigned to the macromolecule as well as ligands (Gasteiger for the ligands and Kollman for the protein) (26).

The regions of interest of the enzyme were defined by considering Cartesian chart 20.5, 3.5 and 20.45 as the central of a grid size of 42, 42 and 56 points in X, Y and Z axises. Ile557, Ile566, Ile572, Ile515, Phe576, Leu770 and Ile773 were selected flexible. The docking parameter files were generated using Genetic Algorithm and Local Search Parameters (GALS) while number of generations was set to 256. The 256 docked complexes were clustered with a root-mean-square deviation tolerance of 0.2 Å. The program generated 256 compound **1a-f** and **2a-f** docked conformers corresponding to the lowest-energy structures. After docking procedure, docking results were submitted to DS visualize (27) for further evaluations.

***SLO screening assay***

Linoleic acid and two assay solutions (A and B) were prepared in advance. Solution A was 50 mM DMAB in a l00 mM phosphate buffer (pH 7.0). Solution B was a mixture of l0 mM MBTH (3 mL), hemoglobin (5 mg/mL, 3 mL) in 50 mM phosphate buffer at pH 5.0 (25 mL). A linoleic acid solution was prepared by mixing 5 mg of linoleic acid with 0.5 mL ethanol and then diluting with KOH 100 mM to a final volume of 5 mL. In the standard assay, the sample in ethanol (25 µL), SLO (4000 units/mL in 50 mM phosphate buffer pH 7.0; 25 µL) and phosphate buffer pH 7.0 (50 mM; 900 µL) were mixed in a test tube and preincubation was carried out for 5 min at room temperature. A control test was done with the same volume of ethanol. After the pre-incubation, linoleic acid solution (50 µL) was added to start the peroxidation reaction, and, 7 min later, solution A (270 µL) and then solution B (130 µL) was added to start the color formation. 5 min later, 200 µL of a 2% SDS solution was added to terminate the reaction. The absorbance at 598 nm was compared with control test. The IC_50_ results are outlined in Table 1.

## Results

**Figure 1:**
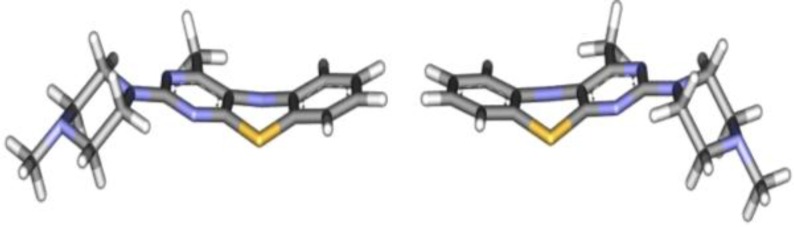
Two enantiomers of compound 2e derived from structural optimization

Structural optimization of the more stable conformers of the mentioned pyrimidobenzothiazines, revealed that for each structure two enantiomers are prone to exist (Figure 1). It can be argued that the presence of these configurations arises from slight bending in the plane of the thiazine ring. In these structures the bond angle between sulfur and two of its adjacent carbon atoms are out-of- plane, bending downwards, which ultimately produces an unsymmetrical structure. It is well documented that the lower the tendency of sulfur pi orbital to participate in sp^3^ hybridization, the more the bending; and because of its electron donating and reducing properties inhibitory role of the sulfur atom increases in thiazine ring. It has also been proved that in the process of the enzyme inhibition, the sulfur atom undergoes oxidation (14).

**Figure 2:**
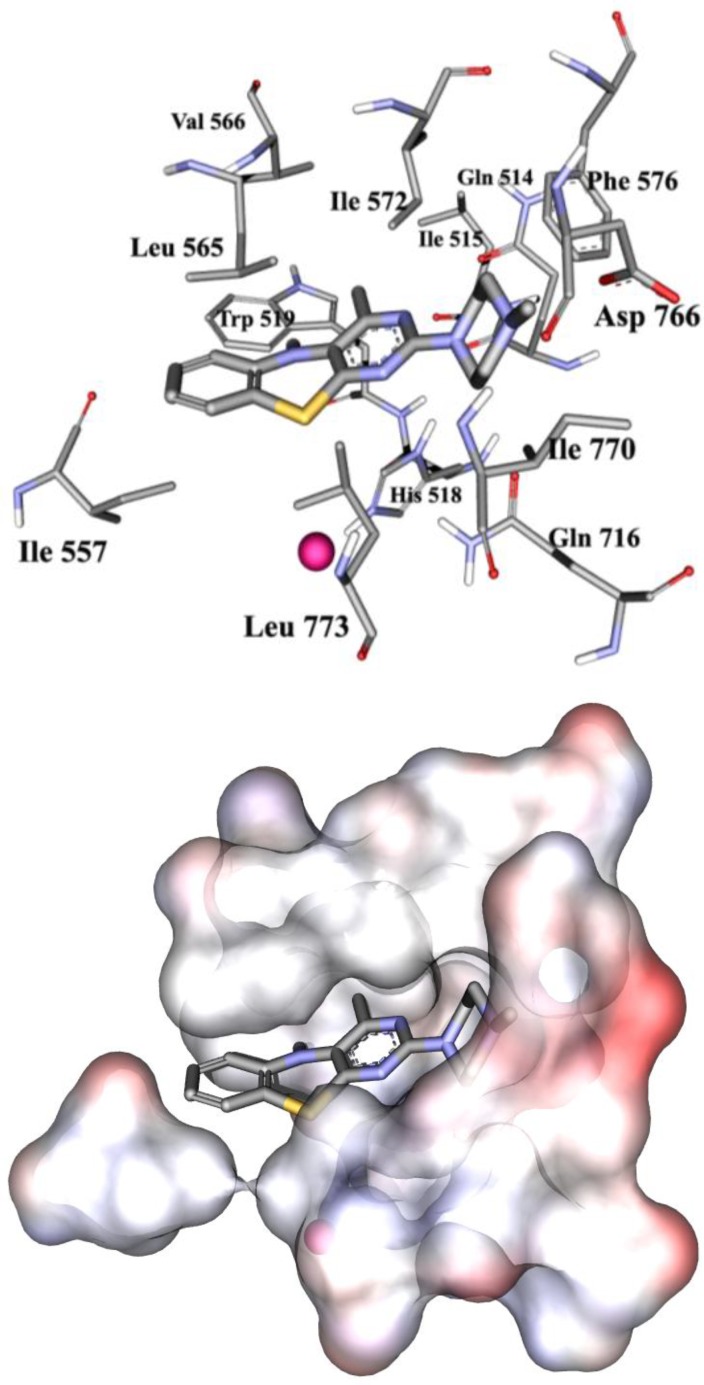
Consensus structure of 2e in the active site pocket of SLO in stick (above) and solvent surface (below) views. Fe atom is distinguished by pink bull. Carbon, oxygen, nitrogen, sulfur and hydrogen atoms are distinguished by gray, red, blue, yellow and white color respectively

The optimized three dimensional structures of compounds **1a-f** and **2a-f** were docked into the active site of the SLO using Auto dock Tools software. The side chain of the hydrophobic amino acids Ile515, Ile557, Leu565, Ile566, Ile572, Phe576, Leu770 and Ile773 which have had direct contact to the substrate in the active site pocket of the enzyme were considered to be flexible. In the docking process, for every compound 256 docked models were obtained among them 25 to 45 percent belongs to a cluster with the sulfur atom oriented vertically towards the Iron atom which is attached to the enzyme. In the software the estimated free bonding energy (∆G_b_) and inhibitory constant (Ki) of each docked model was calculated and outputted as Kcal/mol and mol/L respectively (Table 1). Among the two enantiomers of each compound, the ones with the plane of pyrimidobenzothiazine bending downwards and the sulfur atom in-front with the amine substituent on the right side of the molecule (Figure 1: right) were the responsible models for forming the aforementioned conformation in the active site pocket. The thiazine ring was fixed with two amino acids Leu565, Leu773 and the substituents of the pyrimidine ring were surrounded by the pocket of the amino acids Leu770, Val769, Asp766, Gln716, Phe576, Ile572 and Gln514 (Figure 2).

It is interesting to note that the aforementioned amino acids with the exception of Val769 and Phe576 are conserved (18, 19). In the selected cluster for every docked substituent, the model with the least inhibitory constant (K_i_) was chosen as “consensus structure” to study its structure-activity relationship (Figure 2). The inhibitory activity of compounds **1a-f**, **2a-f** towards SLO enzyme was determined by measuring the amount of peroxide formation. In this method, with the help of a spectrophotometer and by using a mixture of MBTH (3-methyl-2-benzothiazolonhydrazone) and DMAB (3-dimethylaminobenzoic acid) indicators in the presence of hemoglobin at the wavelength of 598 nm, the peroxide concentration was determined (16).

**Table 1:** Docking processing data, enzyme inhibitory assessment and E_HOMO_ and E_LUMO_ of consensus structures (the IC_50_ values are given as mean ± SD)

Compound	∆G_b_	K_i _(fM)	IC_50 _(μM)	E_HOMO _(ev)	E_LUMO _(ev)
1a	-16.56	726	363.1 ± 8.1	-7.053	2.761
1b	-17.28	215.62	203.8 ± 6.4	-7.384	2.689
1c	-17.67	110.85	154.9 ± 3.1	-7.432	2.625
1d	-16.26	1210	536.5 ± 8.8	-7.366	2.612
1e	-17.97	66.82	87.7 ± 2.1	-7.024	2.762
1f	-17.88	77.66	124.8 ± 2.8	-7.125	2.763
2a	-17.55	135.73	197.1 ± 2.4	-7.143	2.825
2b	-17.70	109.94	125 ± 2.1	-7.373	2.742
2c	-17.92	72.97	107.6 ± 2.1	-7.127	2.799
2d	-16.01	1840	395.1 ± 5.1	-7.194	2.639
2e	-18.49	28.04	21.2 ± 1.1	-7.067	2.790
2f	-18.58	24.04	40.7 ± 1.3	-7.009	2.793

## Discussion

Considering the data of Table 1 we can see among the two series of the synthetic compounds the lowest IC_50_ and Ki belong to **1e** and **2e **which have had the N-methyl piperazine moiety. This might be due to the hydrogen bonding formation between protonated tertiary terminal amine and carboxylate portion of conserved Asp766. To prove the hypothesis the inhibitory activity of **1e** and **2e** in comparison with **1c** and **2c** was performed at higher pH (pH = 8.3 versus pH = 7.0). The results showed that the IC_50_ of **1e** and **2e** increase to 129.2 and 39.2 µM while it was nearly unchanged for **1c** and **2c** which have had methyl piperidine moiety. This behavior originated from protonation decrease of N-methyl piperazine moieties at higher pH. Also it is seen that by changing N-methyl piperazine to N-ethyl piperazine (increase of steric hindrance) the IC_50_ value increases. For further proof, the bulker analog **2g** with N-phenyl piperazine moiety and lower protonation potency was tested for SLO inhibitory activity. The IC_50_ value of **2g** was 182 µM which could be another witness for the protonation assertion.

**Figure 3:**
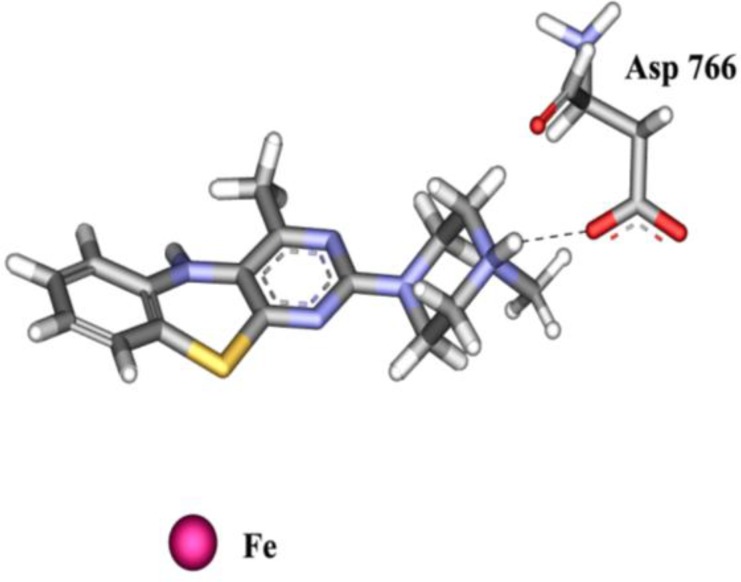
3D view of hydrogen bond between protonated N-methyl piperazine moiety of compound 2e and carboxylate moiety of Asp766

**Figure 4:**
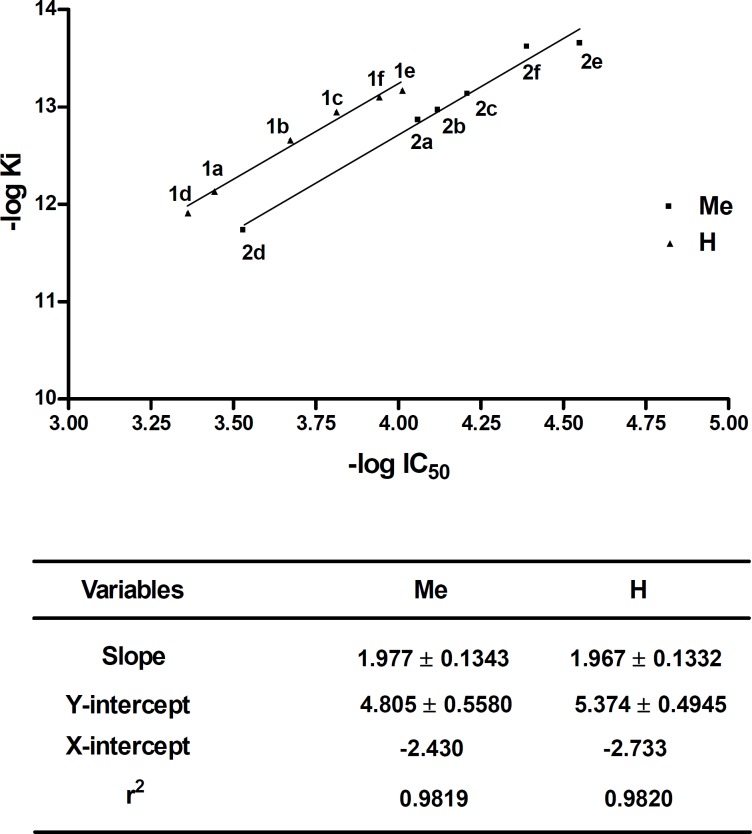
Diagram of (- log Ki) versus (- log IC50) for compounds 1a-f and 2a-f and the relevant data obtained from this diagram. The data of 4-methyl and 4-H pyrimidobenzothiazine analogs are distinguished by Me and H

Finally, the linear dependence of the inhibitory constants (K_i_) of the consensus structures versus the results obtained from the enzyme assay (IC_50_) was studied and is plotted graphically (    Figure 4 ). As it can be seen in     Figure 4  linear plots are obtained with the same gradient but different Y-intercept. This diagram shows that by using molecular simulating software such as Auto dock Tools for pyrimidobenzothiazines, we can predict the inhibitory activity of these substituents by changing the substitution at position 2 providing that the size of the substituents does not change dramatically. On the other hand, these plots clearly show the importance of the methyl substituent at position 4. As can be seen, by putting a methyl substituent at this position, the inhibitory activity increases dramatically which indicate the inhibitory potential of position 4 of the pyrimidobenzothiazines. Previously we showed that the methyl group can fill the hydrophobic cavity formed by Leu515, Trp519, Val566 and Ile572 side chains and it might be one of the reasons of increase in inhibitory activity (15).

In a parallel investigation, the fluctuation in the energy state of HOMO was calculated using single point calculations for a hypothetical reaction concerning the transformation of the consensus structure 4-Me to 4-H, which was followed by using the following relation to obtain the equilibrium constant (K) for the hypothetical process:

∆G^o^ = ∆H^o^ - T∆S^o^

∆S^o^ ~ 0 ΔG^o^ = ∆H^o^ ∆G^o^ = ∆E_HOMO_ = -2.3RTlogK

logK = -ΔE_HOMO_/2.3RT

ΔE_HOMO_ = E_HOMO2_ – E_HOMO1_


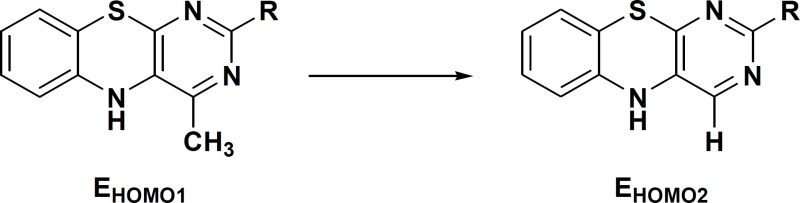


It is interesting that the average of the aforementioned calculated K (K = 0.655) is closed to Y-intercept difference (**5.374 - 4.805 = 0.5690**) of the diagram in     Figure 4 .

By adding the average equilibrium constants to the Y-intercept of new 4-Me analogs, the following new linear plot was obtained. It seems that by changing the substituents at position 2, different analogs of 4-H and 4-Me will be obtained whose their inhibitory activity can be predicted by obtaining their K_i_ using Auto dock Tools and calculating their IC_50_ from the following equation:

log K_i_ = (2.081 ± 0.0803) log IC_50_ - (4.334 ± 0.3163)

The results of our investigations regarding the previously mentioned derivatives show that any changes in HOMO energy state are as important as molecular docking results. For example, if we want to predict the inhibitory activity of a new derivative containing a bulkier substituent like ethyl group at position 4, besides obtaining the inhibitory constant through docking, we must also determine the difference between the HOMO energy states of this derivative with its 4-H analogs.

**Figure 5:**
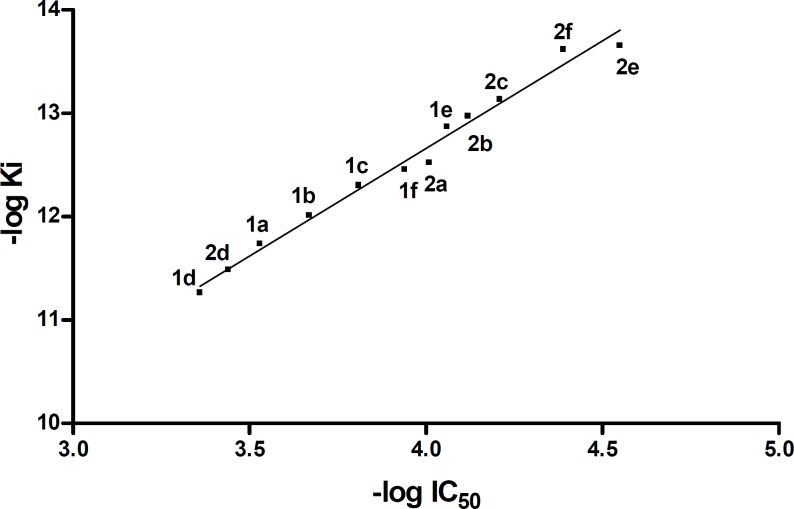
Diagram of (- log K_i_) versus (- log IC_50_) for compounds 1a-f and 2a-f

## Conclusion

Our molecular modeling studies shows that by using more flexible amino acids during the docking process, more rational results can be obtained. The method of measuring the lipoxygenase activity is also of prime importance for the study of structure activity relationship. The reason behind this argument is based on a comparison that we can make between the results of our previous and present investigations. In our previous study, the enzyme activity was measured by considering the formation of the diene at 234 nm, while we based our recent study on measuring the amount of the peroxide formed at 598 nm. In the previous method, due to the slight solubility of some derivatives and turbidity of their solutions, the error was marginally high in the range of UV wavelength, which had a negative effect on the results of enzyme activity. Furthermore, in the docking investigation, we found that amino acids in the active site of the enzyme were not flexible which reduce the chance of finding a suitable binding space for the inhibitor. Because of these two problems, in our previous investigation we did not get a suitable linear relationship between the results of molecular docking and of enzyme activity. We believe our recent investigation is more comprehensive than our previously reported study.
